# Prospective multicenter study evaluating safety, tolerability and metabolism of niraparib as maintenance for ovarian cancer in women of African ancestry

**DOI:** 10.3389/fonc.2026.1976278

**Published:** 2026-09-24

**Authors:** Matthew Schlumbrecht, Bala Audu, Ahmed Hamidu, Aisha Mustapha, Adekunle Oguntayo, Anisah Yahya, Abubakar Bello, Raleigh Butler, Darron Halliday, Saida Bowe, Scott Jordan, Karina Hew, Andre Pinto, R. Patricia Castillo, Isildinha M. Reis, Sophia George

**Affiliations:** 1 Sylvester Comprehensive Cancer Center, Miami, FL, United States; 2 Department of Obstetrics, Gynecology, and Reproductive Sciences, University of Miami Miller School of Medicine, Miami, FL, United States; 3 Federal University of Health Sciences Azare (FUHSA), Azare, Bauchi State, Nigeria; 4 Ahmadu Bello University Teaching Hospital (ABUTH), Zaria, Kaduna State, Nigeria; 5 University of the West Indies-Nassau, Nassau, Bahamas; 6 Broward Health, Fort Lauderdale, FL, United States; 7 Department of Obstetrics and Gynecology, University of Florida-Jacksonville, Jacksonville, FL, United States; 8 Department of Pathology, University of Miami Miller School of Medicine, Miami, FL, United States; 9 Department of Radiology, University of Miami Miller School of Medicine, Miami, FL, United States; 10 Division of Biostatistics and Bioinformatics, Department of Public Health Sciences, University of Miami Miller School of Medicine, Miami, FL, United States

**Keywords:** African ancestry, clinical trial, niraparib, ovarian cancer, PARP inhibitor

## Abstract

Large-scale clinical studies investigating the safety and efficacy of novel therapeutics in ovarian cancer rarely include Black women or women of African ancestry. The sampled population in the PRIMA study was only 1.6% Black. Our objective is to evaluate the safety and tolerability of maintenance niraparib treatment in women of African ancestry. We hypothesize that the Poly (ADP-ribose) polymerase inhibitor (PARP) niraparib administered at standard doses in a population of African ancestry may demonstrate different safety and efficacy profiles to those in the PRIMA trial who were of primarily European ancestry. We will conduct a prospective, single-arm, multicenter, international study of 70 women of African ancestry designed to evaluate the safety and efficacy of niraparib maintenance therapy following completion of primary treatment for epithelial ovarian cancer. Study sites will include the United States, Bahamas, and Nigeria. Eligible patients must have histologically confirmed high-grade serous or endometrioid ovarian carcinoma, completed primary treatment for newly diagnosed stage III or IV ovarian, fallopian tube, or primary peritoneal carcinoma (surgery and chemotherapy) with partial or complete response, and self-identify as Black or African or Afro-Caribbean. The primary endpoint is the proportion of women with any grade 3 or higher adverse event. Secondary endpoints and translational endpoints will include progression free survival, pharmacokinetics, quality of life assessments, and biomarkers of PARP inhibitor resistance and metabolism.

**Clinical Trial Registration:**

https://clinicaltrials.gov/study/NCT06412120, identifier NCT06412120.

## Background

### Ovarian cancer in Black women

Ovarian cancer is the 5th leading cause of cancer death in the United States (1). High- grade serous carcinoma is the most common histotype. Twenty to 25% of women diagnosed with high-grade serous carcinoma have a germline mutation in the homologous recombination deoxyribonucleic acid (DNA) repair pathway (most commonly *BRCA1/2*, *PALB2*, *ATM*, *RAD51C/D*), and about 50% of sporadic tumors have mutations or epigenetic silencing of these genes (2). Black women, when matched for stage, tumor grade, and treatment, have disproportionately worse responses to platinum-based chemotherapy when compared to their White counterparts (3). Although Black women have systematically been undertested for germline and somatic mutations (4), we and others have demonstrated higher than expected rates (10%–25%) of germline *BRCA1* and *BRCA2* mutations in Black patients with breast and ovarian cancer (4, 5). Within The Cancer Genome Atlas (TCGA) ovarian pan cancer data, the largest genomics study to date of ovarian cancer, only 38/592 are confirmed to be of African descent (2). Furthermore, large-scale clinical studies investigating the safety and efficacy of novel therapeutics in ovarian cancer rarely include Black women. For example, the sampled population in the PRIMA study was only 1.6% Black while the sampled population in the NOVA study was only 1.3% (6, 7). Therefore, there is a need to understand the underlying contributions of genetics with respect to disease response to bridge this health disparities gap.

There are more than 21 million Black women in the US (8). Over 10% of the US Black population include African and Caribbean born immigrants. Black immigrants and their children make up 18% of the Black American population (9). Prospective and retrospective studies of West African breast cancer patients from Nigeria show that ~14% had a germline (inherited) deleterious mutation in homologous recombination repair genes, which are responsible for hereditary breast and ovarian cancer syndrome (10). Genetic analysis of tumors from women diagnosed with breast cancer in the Caribbean islands of The Bahamas and Trinidad and Tobago revealed that 10–25% have genetic markers of hereditary breast and ovarian cancer syndrome compared to 5–10% in the general US population (5). Worse overall survival (OS) in Black women and the lack of known side effects/response to poly (adenosine diphosphate [ADP]-ribose) (PARP) inhibitors in this population, require us to pursue these much-needed data for Black patients with ovarian cancer across a range of geographies and backgrounds.

### Ancestry-informed pharmacogenomics, drug dosing, and trial eligibility

Pharmacogenomic-based, personalized dosing recommendations are increasingly recognized as a method to attenuate treatment-related side effects and ensure achievement of serum therapeutic range (11). The importance of this finding specifically for women of African ancestry is increasingly recognized. A significant amount of data have emerged about cytochrome *P450* alleles in Black individuals that are predictive of warfarin metabolism and would drive primary dose modifications (11). Individuals with the CYP2C9*5 allele, which is found almost exclusively in populations of African ancestry, metabolize the drug much more slowly and are at higher risk for overdose. As such, primary dose modifications by up to 30% are required for patients with this allele to avoid toxicity (12).

Country-level investigations of pharmacogenetic-based dosing, also considering ancestry, have demonstrated that these findings are applicable to medications beyond just warfarin. A study in Zimbabwe illustrated that there were high frequencies of African-specific cytochrome *P450* variants which would affect drug metabolism of the most prescribed medications purchased by the state, including antibiotics, anti-epileptics, and chemotherapy agents. The authors suggested that clinicians caring for this population should utilize pharmacogenomics when prescribing new medications entering the market in this region (13). These results all suggest that population-centered approaches to pharmacogenomics, which at present do not exist for PARP inhibitors, would be useful approaches for improving efficacy and minimizing toxicity.

It has also been demonstrated that Black patients have a much higher proportion of Duffy-null erythrocytes compared to Non-Hispanic White patients (66% vs. <1%) (14). This predisposes them to a 40% lower absolute neutrophil count (ANC) (15). In a recent report, it was shown that ANC-based eligibility criteria disproportionately exclude Black patients from participating in clinical trials (16). For those whose baseline ANC may be low, but sufficient for treatment, it may potentially predispose to early treatment discontinuation and/or dose modifications that may not have been necessary had Duffy status been considered.

### Niraparib as maintenance therapy following primary ovarian cancer treatment

The safety of niraparib, an orally available, potent, highly selective PARP1 and PARP2 inhibitor, for the treatment of patients with advanced ovarian cancer following first-line treatment with platinum-based chemotherapy was studied in the PRIMA trial (NCT02655016), a placebo-controlled, double-blind study in which 728 patients in complete or partial response (CR and PR, respectively) received either niraparib or placebo (7). Patients were randomized 2:1 to receive niraparib or matched placebo post-completion of first-line platinum-based chemotherapy plus surgery. Eighty-nine percent of patients in PRIMA were White. Approximately 45% of patients were enrolled in the US or Canada. In the overall population, 65% of patients had stage III disease and 35% had stage IV disease. Among patients who received niraparib, the median duration of treatment was 11.1 months (range: 0.03 to 29 months).

Among the most common grade 3 or higher adverse events in the niraparib group were anemia (in 31.0% of the patients), thrombocytopenia (in 28.7%), and neutropenia (in 12.8%). Dose reductions were conducted in 70.9% of the patients in the niraparib group (7). Serious adverse reactions occurred in 32% of patients receiving niraparib. Serious adverse reactions in >2% of patients were thrombocytopenia (16%), anemia (6%), and small intestinal obstruction (2.9%). Fatal adverse reactions occurred in 0.4% of patients, including intestinal perforation and pleural effusion (1 patient each). Myelodysplastic syndrome or acute myeloid leukemia occurred in 1.2% of patients receiving niraparib. Adverse reactions led to dose reduction or interruption in 80% of patients, most frequently from thrombocytopenia (56%), anemia (33%), and neutropenia (20%). Permanent discontinuation due to adverse reactions occurred in 12% of patients who received niraparib. Adverse reactions resulting in permanent discontinuation in >1% of patients who received niraparib included thrombocytopenia (3.7%), anemia (1.9%), and nausea and neutropenia (1.2% each).

PRIMA demonstrated a statistically significant improvement in progression free survival for patients randomized to niraparib as compared with placebo in the homologous recombination deficiency (hazard ratio [HR] = 0.43; 95% confidence interval [CI]: 0.31 to 0.59; p-value <0.0001) and overall population (HR = 0.62; 95% CI: 0.50 to 0.76; p-value <0.0001). In exploratory subgroup analyses of patients who were administered a starting dose of niraparib or matched placebo based on baseline weight or platelet count, the HR for progression free survival was 0.39 (95% CI: 0.22, 0.72) in the homologous recombination deficient subgroup (n = 130) and 0.68 (95% CI: 0.48, 0.97) in the overall population (n = 258).

## Methods

### Trial design

This is a prospective, single-arm, multicenter, international study of women of African ancestry designed to evaluate the safety and efficacy of niraparib maintenance therapy following completion of primary treatment for high-grade serous or high-grade endometrioid epithelial ovarian, fallopian-tube, and primary peritoneal carcinoma. All patients will receive single agent niraparib with a starting dose of 300mg daily (modified to 200mg daily if weight <77kg or platelets <150,000), with protocol treatment mirroring that of the PRIMA and PRIME studies (7, 17). Patients will be treated for 24 months, or until there is suspicion of recurrence by physician assessment using clinical or biochemical indicators at which point imaging will be performed. Assessment of disease progression will be determined by Response Evaluation Criteria in Solid Tumors (RECIST) v1.1. A blinded independent central review of all imaging will be conducted to independently confirm progression. Funding for this trial has been provided by GSK, and the trial is under registration number NCT06412120. Ethics approval was granted by University of Miami Institutional Review Board (Protocol 20230316).

### Participants

Eligible participants are women who self-identify as Black, African and/or Afro-Caribbean, with newly diagnosed stage III or IV high-grade serous or high-grade endometrioid carcinoma of the ovary, with complete or partial response. Women will be recruited from five sites globally to capture a representative population of Black women: United States (Miami, FL; Fort Lauderdale, FL; and Jacksonville, FL), The Bahamas (Nassau), and Nigeria (Zaria, Kaduna State). Full inclusion and exclusion criteria are shown in **Table 1**.

**Table 1:** Eligibility criteria.

Inclusion criteria	Exclusion criteria
Participant must be female ≥18 years of age, able to understand study procedures, and agree to participate in the study by providing written informed consent. Self-identify as Black. Please note that individuals who identify as Latino are eligible to participate so long as they also self-identify as Black. Participant has completed adjuvant treatment for newly diagnosed stage III or IV ovarian, fallopian tube, or primary peritoneal cancer according to the International Federation of Gynecology and Obstetrics staging criteria. Participant must have high-grade serous or high-grade endometrioid histology. Participant must provide saliva and/or blood specimens for assessment of germline mutation(s) in the Fanconi Anemia pathway Participant must provide formalin-fixed, paraffin-embedded (FFPE) or fresh tumor specimen from initial cytoreductive surgery (primary debulking) or initial pre-treatment core biopsy (if NACT received; tumor obtained from interval cytoreduction acceptable if pre-treatment biopsy not obtained). Participant must have had a complete or partial clinical response to adjuvant treatment as confirmed by CT scan within 8 weeks after completion of the last dose of platinum-based chemotherapy. Participant must have recovered to ≤ Grade 1 in terms of toxicity from prior treatments. Participant must have an Eastern Cooperative Oncology Group (ECOG) performance status 0 – 2. Participant must not have any known contraindication or hypersensitivity to niraparib or any of its excipients. Participants must be considered candidates for maintenance niraparib therapy by their treating physician. Participants should have adequate organ function as defined below: o Platelets ≥ 100 platelets × 10^9^/L o Hemoglobin ≥ 9 g/dL or 5.6 mmol/L o Aspartate transaminase (AST) and alanine transaminase (ALT) ≤2.5 × upper limit of normal (ULN), <5× in patients with known liver metastases o Serum total bilirubin ≤ 1.5 × ULN ◼ 1.5–3.0 × ULN may be included with appropriate starting dose adjustment to 200 mg daily. o Creatinine <1.5 × ULN or estimated glomerular filtration rate (eGFR) ≥50 mL/min by Cockcroft-Gault ◼ Depending on scenario, glomerular filtration rate (GFR) 30–49 mL/min can be permissible. Patients with known human immunodeficiency virus (HIV) are allowed if they meet all the following criteria: o Cluster of differentiation 4 (CD4) ≥350/μL and viral load <400 copies/mL o No history of acquired immunodeficiency syndrome (AIDS)–defining opportunistic infections within 12 months before enrollment o No history of HIV-associated malignancy for the past 5 years. Concurrent antiretroviral therapy as per the most current National Institutes of Health (NIH) Guidelines for the Use of Antiretroviral Agents in Adults and Adolescents with HIV started >4 weeks before study enrollment. A female participant is eligible to participate if she is not pregnant or breastfeeding Participant must agree to complete HRQoL and PRO measures throughout the study period.	Any of the following histologies: low-grade serous carcinoma, grade 1 or 2 endometrioid adenocarcinoma, clear cell, mucinous, transitional cell, carcinosarcoma, undifferentiated, dedifferentiated Any known history or current diagnosis of MDS or AML Primary progressive, platinum-refractory disease Participant is at an increased risk of bleeding due to concurrent conditions (e.g., major injuries or major surgery within the past 28 days before start of study treatment). Current diagnosis of platelet disorder (e.g., thrombotic thrombocytopenic purpura (TTP), immune thrombocytopenia [ITP]) Inability to swallow orally administered medication or has a gastrointestinal disorder likely to interfere with absorption of the study medication Participants that have systolic blood pressure (SBP) >140 mmHg or diastolic blood pressure (DBP) >90 mmHg. Active second primary malignancy Participant is pregnant, currently breastfeeding an infant, or expecting to conceive children while receiving study treatment and/or for up to 180 days after the last dose of study treatment. Participant has received a live vaccine within 30 days of planned start of study therapy. Coronavirus disease 2019 (COVID-19) vaccines that do not contain live viruses are allowed. Participant has received a transfusion (platelets or red blood cells) or colony-stimulating factors (e.g., granulocyte macrophage colony-stimulating factor or recombinant erythropoietin) within 4 weeks before the first dose of study treatment. Participant has had radiotherapy encompassing >20% of the bone marrow within 2 weeks or any radiation therapy within 1 week before Day 1 of protocol therapy. Participant has leptomeningeal disease, carcinomatous meningitis, symptomatic brain metastasis, or radiographic signs of central nervous system hemorrhage. Participant has current active pneumonitis or any history of pneumonitis requiring steroids (any dose) or immunomodulatory treatment within 90 days of planned start of the study. Participants with active hepatitis B or C infection. Patient with prior history of PRES. Patients with impaired decision-making capacity. Participants have high medical risk due to a serious, uncontrolled medical disorder; non-malignant systemic disease; or active, uncontrolled infection. Examples include, but are not limited to, uncontrolled ventricular arrhythmia, recent (within 90 days) myocardial infarction, uncontrolled major seizure disorder, unstable spinal cord compression, superior vena cava syndrome, active uncontrolled coagulopathy, bleeding disorder, or any psychiatric disorder that prohibits obtaining informed consent. Patient is currently receiving one or more cytotoxic, hormonal, or other medications to treat their cancer.

NACT, Neoadjuvant Chemotherapy; HRQoL, Health Related Quality of Life; PRO, Patient Reported Outcomes; MDS, myelodysplastic syndrome; AML, acute myeloid leukemia; PRES, posterior reversible encephalopathy syndrome.

### Primary endpoints

The primary endpoint is the proportion of women with any one grade 3 or higher adverse event as measured using the National Cancer Institute (NCI) Common Terminology for Adverse Events (CTCAE) version 5.0 of any treatment attribution and treatment-related for the following eleven most common adverse events previously reported in the PRIMA study (7): anemia, nausea, thrombocytopenia, constipation, fatigue, neutropenia, headache, insomnia, vomiting, abdominal pain, and hypertension.

### Secondary endpoints

Proportions of women with: (1) Any grade and grade 3 or higher treatment-related adverse event, (2) serious adverse event of any treatment attribution, (3) treatment-related adverse events or serious adverse events leading to treatment discontinuation, dose reduction, dose interruption, and death. Further, recurrence-free survival, progression-free survival, trends in serum cancer antigen 125 (CA125) levels, pharmacokinetics and pharmacogenomics of niraparib, and health related quality of life measures utilizing the Functional Assessment of Cancer Therapy-Ovarian Symptom Index and the Functional Assessment of Cancer Therapy-Item GP5, will be characterized.

### Translational endpoints

The translational aims are to identify biomarkers of PARP inhibitor resistance, metabolism and response. We will ascertain the prevalence of somatic homologous recombination deficiency and homologous recombination proficiency as well as germline mutations in this population. We will calculate the proportion of single nucleotide polymorphisms indicative of differential drug metabolic activity (pharmacogenomics) to determine if any are suitable for further study as biomarkers of altered pharmacokinetics and/or toxicity. Circulating tumor DNA will be measured across the study interval to identify early trends which may suggest recurrence.

### Sample size

The study population was defined as 70 patients for analysis of the primary endpoint based on the accrual capacity of the clinical sites and the desire to complete this study within 5 years, including an expected accrual within 2 years and a minimum study participation of 3 years from start of maintenance therapy. This sample size will provide good precision for estimation of treatment-related grade ≥3 rate of a specific adverse event. Using the Clopper-Pearson method, for a possibly observed rate of 30%, the corresponding 95% confidence interval (CI) would be 19.6% to 42.1% (width=22.5%); for a smaller rate of 15%, the 95% CI would be 7.6% to 25.5% (width=17.9%).

### Early stopping rules

Early stopping (suspension and possibly termination) of the study will be considered if there is evidence that the proportion of patients experiencing unacceptable toxicity exceeds 35%. Specifically, we suggest as a guideline for early termination a posterior probability (based on Bayesian probabilities) of 80% or higher such that the rate of unacceptable toxicity exceeds 35%. Posterior probabilities used to derive the following table are calculated under a prior beta distribution with parameters b1 = 0.35 and b2 = 0.65, which corresponds to an expected rate of 35% based on very limited information. We also set a minimum of 12 patients before the early stopping rules apply.

## Analysis of results

The primary endpoint is safety, and will be summarized in the safety population, defined as all participants who received at least one dose of niraparib. For each of the 11 most common reported adverse events (anemia, nausea, thrombocytopenia, constipation, fatigue, neutropenia, headache, insomnia, vomiting, abdominal pain, and hypertension), the proportion of patients with grade 3 or higher adverse events or severe adverse events, both of any treatment attribution and treatment-related, will be reported with corresponding 95% confidence intervals (CIs). Proportions of women with (1) Any grade and grade 3 or higher adverse event regardless of treatment attribution, (2) Any grade and grade 3 or higher treatment-related adverse event, (3) serious adverse event of any treatment attribution, and (4) adverse event/serious adverse event leading to treatment discontinuation, dose reduction, dose interruption, and death will also be reported.

Recurrence-free survival will be estimated using the Kaplan-Meier method. The log-rank test and/or Cox proportional hazards models will be used to assess predictors of survival. CA125 will be measured at baseline and monthly over time. The percentage change from baseline will be analyzed using mixed effects modeling for longitudinal data.

Ovarian cancer-specific patient reported health related quality of life outcomes will be measured. The change or the percentage change from baseline will be analyzed using mixed effects modeling for longitudinal data, which considers missing data and within subject correlations over time. Mean score, mean change or mean % change with corresponding standard error (SE) or 95% confidence intervals (CIs) over time will be determined.

To ascertain population structure, we will genotype study participants and infer genetic ancestry using Illumina’s Infinium Human Global Diversity Array with Enhanced PGx-8 v1.0. We will use the genotype data from the Global Diversity Array, which contains over 6,000 single nucleotide polymorphisms identified by the ClinVar, Clinical Pharmacogenetics (PGx) Implementation Consortium (CPIC, [cpicpgx.org]) and the PharmGKB database (www.pharmgkb.org), to correlate with pharmacokinetic measurements. Analyses will be adjusted for age, sex, baseline pharmacokinetics, and principal components for ancestry. Statistical significance will be defined as a Bonferroni corrected p-value for the number of independent single nucleotide polymorphisms (~p<2x10-6).

## Discussion

PARP inhibitor administration following primary treatment for high-grade ovarian, fallopian tube, and primary peritoneal carcinoma has completely changed the treatment landscape of this disease, translating into a more favorable disease trajectory for many women. Unfortunately, the large, Phase III studies demonstrating the efficacy of these new medications had very few participants of African ancestry. This represents a large gap in knowledge as the safety and efficacy of PARP inhibitors is these women is unknown, despite its use in clinical practice.

This study seeks to establish not only how well these medications are tolerated in Black women from across the globe, but will determine efficacy, quality of life aberrations, and patterns of serum biomarkers. Importantly, it will establish if current dosing schedules, beyond modifications for weight and platelets, are appropriate in this population. Because our cohort is representative of a diverse population of Black women, including those from West Africa (Nigeria), the Caribbean (The Bahamas), and the United States, the potential for global applicability of these results is substantial. Furthermore, we demonstrate that a multicenter, international clinical trial for gynecologic cancer can be feasibly executed in low to middle income countries when dedicated capacity building is undertaken. We therefore hope that the successful completion of this study will result in the consideration of these regions as sites for clinical trial enrollment in further investigations. Additionally, by introducing novel therapeutics into areas which have limited experience with them, if found efficacious in these populations, there is the potential for advocacy at the governmental level to acquire these drugs or at least open up conversations with industry partners to facilitate expanded access.

The landscape of PARP inhibitors has evolved significantly, as have the indications. Initial prescribing was relatively liberal, especially for niraparib, but in subsequent years additional survival data have translated into more restricted use. While this is an important consideration, it is important to recognize that the data driving current treatment guidelines are based on a primarily European study population. Our study intentionally includes Black women with both homologous recombination deficient and homologous recombination proficient disease to ensure that there are no missed opportunities for use of drug in a population that may derive benefit. Close safety monitoring is planned as we explore a more comprehensive personalized treatment approach in these women.

## Conclusion

In summary, this prospective single-arm study of niraparib for maintenance in women of African ancestry represents a significant opportunity to enhance medication equity across not only race, but geography. The data generated from this investigation has the potential to guide PARP inhibitor use in a population of women at risk, while simultaneously providing a framework for multicenter, multi-national clinical trials across the globe.

## Data Availability

The original contributions presented in the study are included in the article/supplementary material. Further inquiries can be directed to the corresponding author.
